# Modeling receptor flexibility in the structure-based design of KRAS^G12C^ inhibitors

**DOI:** 10.1007/s10822-022-00467-0

**Published:** 2022-08-05

**Authors:** Kai Zhu, Cui Li, Kingsley Y. Wu, Christopher Mohr, Xun Li, Brian Lanman

**Affiliations:** 1 Department of Molecular Engineering, Amgen Inc., One Amgen Center Drive, Thousand Oaks, CA 91320 USA; 2 Department of Medicinal Chemistry, Amgen Inc., One Amgen Center Drive, Thousand Oaks, CA 91320 USA; 3 Amgen Asia R&D Center, 13th Floor, Building No. 2, 4560 Jinke Road, Zhangjiang, Shanghai, 201210 China; 4 Department of Chemistry, University of California, 501 Big Springs Road, Riverside, CA 92521 USA

**Keywords:** Covalent docking, Pose prediction, Binding affinity, Free energy perturbation (FEP), Switch-II pocket

## Abstract

KRAS has long been referred to as an ‘undruggable’ target due to its high affinity for its cognate ligands (GDP and GTP) and its lack of readily exploited allosteric binding pockets. Recent progress in the development of covalent inhibitors of KRAS^G12C^ has revealed that occupancy of an allosteric binding site located between the α3-helix and switch-II loop of KRAS^G12C^—sometimes referred to as the ‘switch-II pocket’—holds great potential in the design of direct inhibitors of KRAS^G12C^. In studying diverse switch-II pocket binders during the development of sotorasib (AMG 510), the first FDA-approved inhibitor of KRAS^G12C^, we found the dramatic conformational flexibility of the switch-II pocket posing significant challenges toward the structure-based design of inhibitors. Here, we present our computational approaches for dealing with receptor flexibility in the prediction of ligand binding pose and binding affinity. For binding pose prediction, we modified the covalent docking program CovDock to allow for protein conformational mobility. This new docking approach, termed as FlexCovDock, improves success rates from 55 to 89% for binding pose prediction on a dataset of 10 cross-docking cases and has been prospectively validated across diverse ligand chemotypes. For binding affinity prediction, we found standard free energy perturbation (FEP) methods could not adequately handle the significant conformational change of the switch-II loop. We developed a new computational strategy to accelerate conformational transitions through the use of targeted protein mutations. Using this methodology, the mean unsigned error (MUE) of binding affinity prediction were reduced from 1.44 to 0.89 kcal/mol on a set of 14 compounds. These approaches were of significant use in facilitating the structure-based design of KRAS^G12C^ inhibitors and are anticipated to be of further use in the design of covalent (and noncovalent) inhibitors of other conformationally labile protein targets.

**Supplementary Information:**

The online version contains supplementary material available at 10.1007/s10822-022-00467-0.

## Introduction

KRAS is a G-protein that functions as a molecular switch regulating cellular proliferation in growth factor signaling pathways [1]. Mutations in KRAS impair the cycling of KRAS between its GTP-bound active state and GDP-bound inactive state, leading to dysregulated cellular growth and oncogenesis. *KRAS* was identified as one of the first oncogenes in 1982 [2], but extensive research efforts over the following decades failed to provide clinically viable inhibitors of KRAS until the recent approval of sotorasib (**3**; AMG 510) in 2021 [3, 4].

Two factors contributed to the challenge in identifying inhibitors: (1) KRAS binds its native ligands, GDP and GTP, with picomolar affinity and high intracellular concentrations, making competitive inhibition challenging, and (2) other allosteric pockets on KRAS were either incompletely defined or lacked high-affinity ligands, posing challenges to the identification of allosteric inhibitors. In 2013, Shokat and colleagues [5] first reported the X-ray crystal structure of a covalent inhibitor bound to an engineered (‘cysteine-light’) version of the KRAS codon 12 mutant KRAS^G12C^. This inhibitor bound in an allosteric pocket near the GDP binding site that the researchers termed the ‘switch-II pocket’ (and which had previously been referred to as the ‘P2 pocket’ [6] or ‘site 3’ [7]). This discovery, which built on an ongoing resurgence of interest in covalent inhibitors [8], was followed by multiple additional reports of covalent KRAS inhibitors targeting KRAS^G12C^. In addition to sotorasib (**3**), which remains the only clinically approved KRAS^G12C^ inhibitor to date, eleven other covalent KRAS^G12C^ inhibitors targeting the switch-II pocket have now entered human clinical trials [9].

**Fig. 1:**
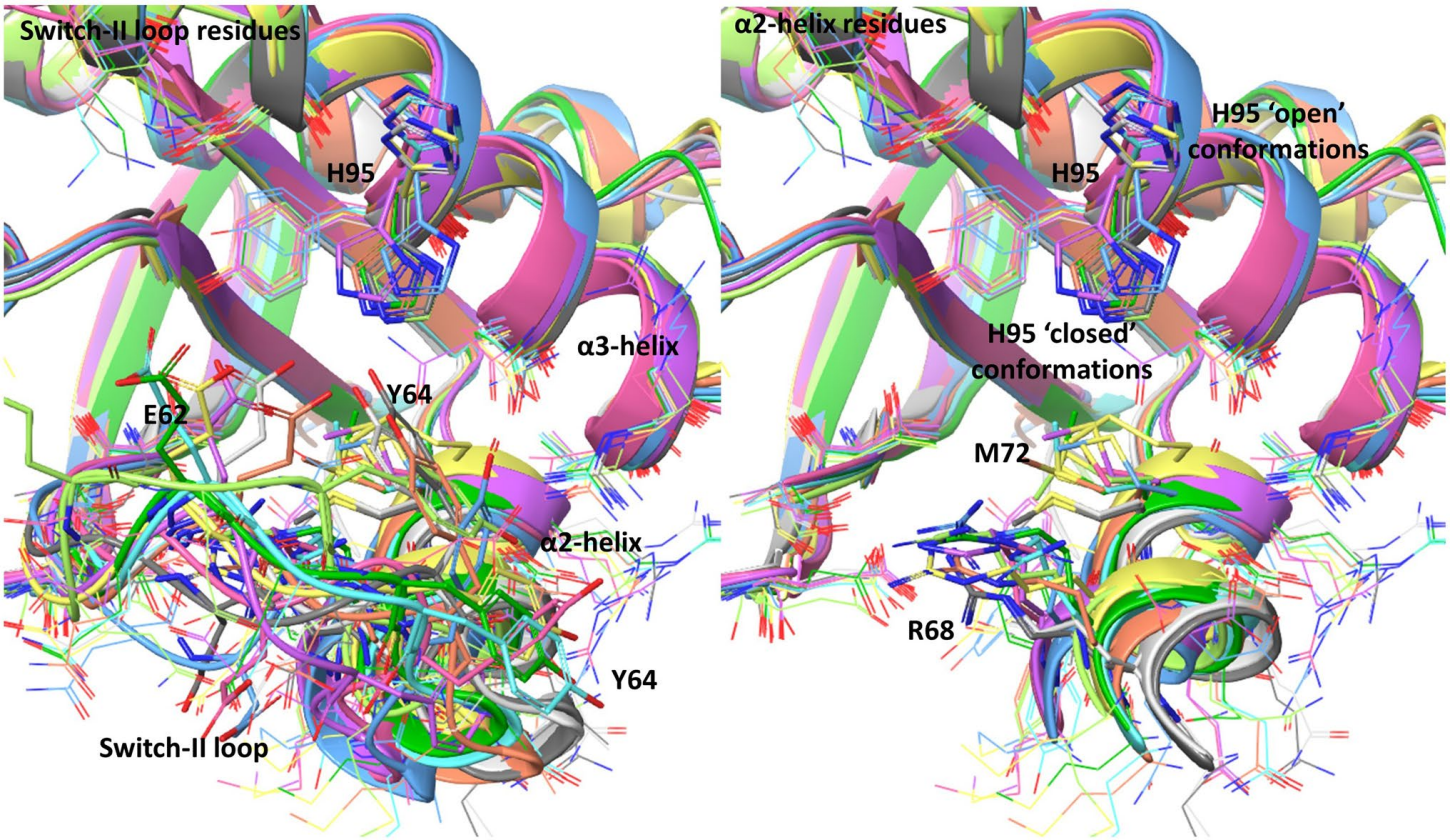
Illustration of flexible residues in the switch-II binding pocket of KRAS^G12C^. Receptor structures (10) from the cross-docking dataset are superimposed. All flexible residues in the FlexCovDock workflow are depicted in wire mode and five blocking residues (labeled) are shown in tube mode. In the right-hand panel, the switch-II loop is removed to reveal the two inside residues, R68 and M72

The switch-II pocket is a shallow pocket between the effector protein-engaging switch-II loop of KRAS (residues A59–Y64), the α2-helix (S65–T74), and the α3-helix (N86–K104, see Fig. 1) [10]. Upon ligand binding, this pocket undergoes significant conformational rearrangement [e.g., increasing in size from 150 to 280 Å^3^ in the case of sotorasib (**3**)]. This conformational change includes significant movement of the switch-II loop, a shift in position of the α2-helix, and side chain rotameric changes in many binding site residues. In work leading to the discovery of sotorasib (**3**), we found that the switch-II binding site proved to be highly flexible, with ligands binding to this pocket producing dozens of conformationally distinct poses, as illustrated in Fig. 1. This conformational flexibility posed a considerable challenge in using protein crystallographic data in the design and optimization of switch-II pocket-targeted covalent inhibitors of KRAS^G12C^.

‘Docking’ is one of the most widely used computational tools for predicting ligand binding poses in drug discovery projects. Most docking methods have been developed to allow for full ligand flexibility in docking into a conformationally rigid receptor pocket. However, such an approach is ill-suited for the computational docking of switch-II pocket ligands to KRAS, as the diverse range of switch-II pocket conformations observed with diverse ligands indicates that significant receptor flexibility must also be considered during docking calculations to ensure that relevant binding poses are identified and evaluated. Incorporation of receptor flexibility remains a challenge at the frontier of next generation docking method development [11, 12].

One common approach to addressing receptor conformational dynamics is ensemble docking, which runs multiple docking calculations with an ensemble of receptor structures [13, 14]. Appropriate structural ensembles are selected to encompass likely receptor conformational changes and can be generated from experimental structures, molecular dynamics simulations, or normal-mode analysis. The key to successful ensemble docking is the proper weighting of each structure to properly reflect its relative population [15]. Another common approach to incorporate receptor flexibility is to model receptor structural changes during the docking process, as is done in docking protocols such as AutoDockFR [16], IFD [17] and IFD-MD [18].

For covalent inhibitors, docking protocols must also deal with the added complexity of accounting for covalent bond formation between the ligand and target protein [19]. Many covalent docking protocols have been developed based on the conventional docking programs. For example, CovDock [20, 21] is a workflow utilizing the Glide docking protocol [22–24] and Prime protein structure refinement [25, 26]; DOCKovalent is a variation of DOCK [27]. AutoDock [28] natively supports covalent inhibitor docking, as do CovalentDock [29] and WIDOCK [30], which are based on AutoDock.

There have been several reports of using covalent docking successfully in the virtual screening and lead optimalization [27, 30–32]. However, to our knowledge, none of these covalent docking methods has the capability to deal with flexible receptors. In this work, we have modified CovDock, which has shown superior accuracy in binding pose prediction [20], to incorporate receptor flexibility, and have successfully used the modified protocol in the design and optimization of covalent inhibitors of KRAS^G12C^.

Beyond simply complicating binding pose prediction, receptor flexibility also significantly complicates the accurate prediction of binding affinities. Several computational approaches have been developed to address this complexity. In docking calculations, ‘docking scores’ are frequently used to rank different poses while also providing an estimate of binding affinity derived from empirical functions capturing various binding energy components such as hydrophobic interactions and hydrogen bonds. Docking scores, however, have been shown to be more successful in predicting binding poses (i.e., ranking different poses for one compound) than in predicting binding affinities (i.e., ranking the relative binding affinities of different compounds) [33].

More accurate binding affinity predictions can often be obtained with advanced computational methods such as MMGB (sometimes termed as MMGBSA) or free energy methods, which provide a better description of binding energetics [34–37]. MMGB incorporates solvent electrostatic interactions into binding energy calculations using the generalized born model, and has been successfully used to re-score docking poses to provide more accurate binding energy predictions [37]. Both docking and MMGB methods rely on a static structure to compute the protein–ligand interactions, while other important contributions such as entropy and structured waters are ignored or treated heuristically.

In recent years, free energy methods, such as free energy perturbation (FEP) and thermodynamic integration (TI) have gained popularity in the industrial setting due to advances in method development and the availability of affordable GPUs, which has enabled calculation at reasonable costs and timescales [38, 39]. These methods use molecular dynamics to simulate the motion of the protein–ligand system in water, and thus naturally account for both receptor flexibility and ligand flexibility. Free energy methods provide a detailed description of all physical forces impacting protein–ligand binding in the framework of classical mechanics and have shown superior accuracy in many systems [34, 35, 38, 39]. Nevertheless, due to the limited time scale of computationally tractable MD simulations, free energy methods can not adequately sample large conformational changes, especially those involving protein backbone movements. This limitation became evident in our study of KRAS switch-II pocket ligands, where inhibitor interactions with the conformationally labile switch-II loop played a key role in ligand–receptor binding affinity. Here, we report a strategy we have designed to overcome this issue and improved the accuracy of FEP calculations involving such challenging ligand–backbone interactions.

In the following sections, we describe our approaches to the modification of the CovDock covalent docking protocol and FEP binding affinity protocol to address these challenges. We describe the construction of a cross-docking database comprising 100 examples generated from 10 structurally diverse KRAS^G12C^ inhibitors with known X-ray crystal structures (3 of which are newly solved for this data set) and describe a method of incorporating receptor flexibility into CovDock to produce a docking protocol we refer to as FlexCovDock. We subsequently describe the characterization of switch-II pocket flexibility, the construction of flexible residue list in FlexCovDock, and the improved performance of FlexCovDock relative to CovDock in binding pose prediction.

To assess the performance of various binding affinity prediction methods, we collected three groups of compounds that engaged in distinct binding interactions with the switch-II pocket, including one group of compounds that have not been published previously. These groups demonstrate distinct ligand flexibility profiles and receptor conformations and serve to illustrate the relative strengths and limitations of binding affinity prediction using docking score, MMGB, and FEP methodology. Analyzing these sets of compounds, we show that conventional FEP methodology works well on relatively rigid receptor pocket, but cannot adequately handle switch-II loop conformational flexibility. A loop mutation strategy is then shown to accelerate the conformational transitions and improve the accuracy of FEP binding energy calculations.

## Materials and methods

### Cross-docking data set

Cross-docking is a process that computationally fits a compound into a receptor structure solved using another compound as a binding ligand. Self-docking, in contrast, describes the process of docking a native ligand back into its experimentally determined crystal structure after having removed the crystallography resolved ligand. Cross-docking, therefore, represents the typical scenario encountered in discovery research where a ligand is docked into an experimentally determined protein structure (obtained using a different ligand) with the aim of generating a realistic binding pose prediction to support ligand optimization. When the receptor binding pocket is flexible and the new compound is substantially different from the native ligand, large conformational changes in the binding pocket can be expected in binding to the new compound. It was this cross-docking challenge (specifically applied to covalent ligands) that we sought to overcome in the development of FlexCovDock methodology. To illustrate the magnitude of these challenges in the context of the KRAS^G12C^ switch-II pocket, we created a cross-docking data set that incorporated ten structurally diverse ligands with crystallographically determined binding poses (see Table 1) and cross- (and self-) docked each of these ligands into each crystallographically determined receptor structure. These ten protein–ligand complexes were selected to cover the wide range of known switch-II binding modes and receptor conformations. Among these ten structures, seven are previously published and three (8DNI, 8DNJ, and 8DNK) are newly reported.

**Table 1:** Protein–ligand structures employed in cross-docking

PDB	5F2E	5V9U	6OIM	6P8X	6UT0
Ligand					
	ARS-853 [40] (1)	ARS-1620 [41] (2)	Sotorasib (AMG 510) [3] (3)	Amgen Cmpd 5 [42] (4)	Adagrasib (MRTX849) [43] (5)

PDB	6T5B	6TAN	8DNI	8DNJ	8DNK
Ligand					
	AstraZeneca Cmpd 25 [44] (6)	Bayer Cmpd 13 [45] (7)	Araxes Cmpd I-1 [46] (8)	AstraZeneca Cmpd 76 [47] (9)	Taiho Cmpd 6 [48] (10)

All structures determined by X-ray crystallography

### FlexCovDock workflow

CovDock is the covalent docking workflow in the Schrödinger Suite that combines Glide small molecule docking and Prime protein structure refinement, which has been described previously in detail [20]. Here, we provide a brief summary as an overview: In the first stage of CovDock docking, the target reactive residue is mutated to alanine, and Glide is then used to dock the target ligand into the binding pocket. In the second stage, the reactive residue is mutated back to its original identity, and its side chain rotamer states are enumerated and combined with differing ligand poses from the Glide docking. The protocol then attempts to form covalent bonds between the various reactive residue side chain rotamers and differing Glide-generated ligand poses, rejecting covalent adducts where proper bond geometry cannot be obtained. In the last stage of the protocol, the resulting candidate covalent adduct poses are clustered, refined, and ranked using the Prime energy function.

In FlexCovDock, two key modifications are made to this workflow: (1) residues that severely block the binding site (as discussed below) are mutated to alanine (alongside the target reactive residue) prior to initial Glide docking. These residues are subsequently mutated back to their original identities after the generation of an ensemble of docked poses; (2) in the Prime refinement stage, a binding site refinement protocol is applied which re-packs the side chains of flexible binding site residues and minimizes all atoms of the ligand and binding site residues. This protocol is available in Prime as a function call “siteopt” and the side chain repacking algorithm has been described in a previous publication [25]. A schematic workflow comparison between CovDock and FlexCovDock is shown in Supplementary Information Fig. S1. For the KRAS^G12C^ switch-II pocket, the choice of ‘blocking’ residues and ‘flexible’ residues is discussed in the Results and Discussion.

### Binding affinity data set

To evaluate binding affinity prediction using docking score, MMGBSA, and FEP, we assembled three sets of compounds (*SAR1–3*) which probe differing regions of the switch-II binding pocket. *SAR1* compounds are taken from Table 1 of Shin, et al. [42], *SAR2* compounds are from Table 2 of Lanman, et al. [3], and *SAR3* compounds are not published previously, which comprise a set of 14 compounds having the same quinazolinone core as sotorasib (**3**) and whose N1-substituents interact directly with the switch-II loop. (Co-crystal structures of all 14 of these compounds have been solved internally.) The structures and experimental binding affinities of these compounds are reported in the Supplementary Information (Table S1–S3). Assay conditions for the determination of experimental binding affinities have been described previously [3, 42]. It should be noted that all compounds in our datasets are irreversible covalent inhibitors. The binding affinities (and IC_50_ values) reported here are not measured at equilibrium but are rather measured at a fixed time point (i.e., five minutes after inhibitor introduction). All compounds within each dataset share the same reactive warhead and are assumed to have approximately the same intrinsic rate of reaction with their target cysteine residues. Thus, differences in binding affinity (at a fixed timepoint) reflect the influence of differing non-covalent protein–ligand interactions (vs. intrinsic warhead reactivity factors) and can be modelled using the various affinity prediction methods employed in this work (e.g., docking score, MMGBSA, and FEP).

### Computational setup

All computations were performed with Schrödinger Suite 2020-2 [49]. MMGB scoring was conducted using the Prime VSGB2.0 model [50] and OPLS3e force field [51]. FEP calculations were performed using FEP+ with the force field OPLS3e. Unless otherwise noted, all FEP+ calculations were performed using default settings and 10 ns of simulation. The FlexCovDock workflow was a modified version of CovDock with the Schrödinger Python API. Prior to calculation, all ligands were prepared using LigPrep, and a single tautomer and charge state was chosen based on PROPKA at pH 7.0. All receptor structures were prepared with Protein Preparation Wizard with default settings.

### X-ray crystallography

A ‘cysteine-light’ mutant construct was used for co-crystallization studies, based on the work of Ostrem et al. [5]. Purified recombinant human KRAS_1–169_^G12C/C51S/C80L/C118S^ protein (untagged) in 20 mM HEPES pH 7.5, 150 mM NaCl was concentrated to 40–50 mg/ml, and added to a twofold molar excess of solid compound dissolved in DMSO. The resulting ligand–protein complex was incubated at room temperature for 16 h on a mixer, and subsequently spin-filtered. LC Mass Spectrometry was performed to determine % conjugation of covalently modified sample.

Co-crystallization was performed using the sitting drop vapor diffusion method. Covalent ligand–protein complex samples were mixed 1:1 with crystallization buffer using a Mosquito® robot (SPT Labtech). Crystals appeared within 1 week at 20 °C. KRAS^G12C^ complexed to compound **8** crystallized in 0.1 M MES pH 6.5, 30% PEG-4000, 1 mM MgCl_2_; compound **9** crystallized in 0.1 M MES pH 6.5, 30% PEG-4000, 1 mM MgCl_2_, 10% EtOH; and compound **10** crystallized in 0.1 M TRIS pH 8.5, 2 M (NH_4_)_2_SO_4_.

All data sets were collected on a Pilatus3 6 M silicon pixel detector at the Advanced Light Source Beamline 5.0.2 at wavelength 1.00000 Å and temperature 100 K. The data were integrated and scaled using either HKL2000 [52] or DIALS [53]. The structures were solved by molecular replacement using Phaser [54] from the CCP4 program suite [55], with an apo-KRAS structure as a search model. The structures were refined using Refmac5 [56], and model building was performed using the graphics program Coot [57]. The ligands were generated using PRODRG [58]. The structure of KRAS_1–169_^G12C/C51S/C80L/C118S^ bound to Mg^+^ GDP and compound **8** was refined to 1.50 Å with an R-factor of 24% and R_free_ of 25%; compound **9** was refined to 1.81 Å with an R-factor of 24% and R_free_ of 29%; compound **10** was refined to 2.23 Å with an R-factor of 18% and R_free_ of 23%.

The atomic coordinates and structure factors have been deposited in the Protein Data Bank respectively (PDB ID codes: 8DNI, 8DNJ, 8DNK). See Supporting Information Table S4 for more details on data collection and refinement statistics.

## Results and discussion

### Characterization of binding site residue flexibility

Figure 1 illustrates the challenge posed by conventional cross-docking of different ligands with differing receptor structures. As shown, superposition of the ten receptor structures from the cross-docking data set reveals dramatically differing backbone and side chain conformations for the switch-II loop residues (Ala59–Tyr64). The adjacent α2-helix (Ser65–Thr74) likewise demonstrates significant conformational shifts across this set of X-ray structures. This conformational flexibility results in markedly different ligand–receptor interactions in differing X-ray structures.

One particularly stark example of this is associated with the conformationally flexible His95 residue. In 2019, Amgen researchers reported that the His95 residue of KRAS could adopt conformation, in which its side chain was oriented away from the switch-II pocket, opening a previously unrecognized ‘cryptic’ pocket [42]. This pocket, which had not previously been exploited by reported covalent KRAS^G12C^ inhibitors, was subsequently leveraged in the design of the covalent inhibitor sotorasib [3]. Sotorasib (**3**) binds to the switch-II pocket region of KRAS by additionally engaging this cryptic sub-pocket, which is revealed in the ‘open’ conformation of His95 (see label, Fig. 1); other ligands, such as ARS-1620 (**2**), bind instead to a switch-II pocket conformation in which the His95 side chain adopts a ‘closed’ conformation (see Fig. 1).

In addition to His95, a range of other switch-II pocket residues demonstrate differing degrees of ligand engagement across the structures included in the cross-docking data set. Within the switch-II loop, Glu63 and Tyr64 are two prominent examples. Tyr64, for example, while frequently solvent-exposed, can sometimes adopt a pocket-facing conformation and engage in π-stacking interactions with the ligand. Glu63, in contrast, has always been observed to be oriented toward the solvent, and its side chain does not make direct contacts with switch-II pocket ligands in any structures.

Within the α2-helix, Arg68 and Met72 represent two-pocket-facing residues whose flexible side chains have been observed to adopt diverse conformations when complexed to differing switch-II pocket ligands, and whose positioning is additionally impacted by conformational changes in the position and rotation of α2-helix. Further highlighting the high conformational mobility of the switch-II loop, it should be noted that switch-II loop conformation can be significantly influenced by crystal packing interactions [10]. Ligand–receptor interactions can differ substantially between different copies of the same binding site within a single crystallographic unit cell. Additionally, the high flexibility of the switch-II loop frequently prevents the assignment of positions for all residues of the switch-II loop in some x-ray structures.

### Blocking residues and flexible residues

The high conformational flexibility of the switch-II pocket posed two chief problems for accurate docking: (1) the need to incorporate multiple switch-II and α2-helix conformations in docking, and (2) the need to accommodate a high degree of side chain flexibility in docking. To successful address these challenges, we found it useful to divide switch-II pocket residues into two classes: (1) residues inside the switch-II pocket that severely impair the cross-docking of another ligand (‘blocking residues’); and (2) flexible peripheral residues that do not severely block the pocket but that can make important ligand–receptor interactions following ligand binding (‘flexible residues’). These residues were initially identified through inspection of the ten structures in the cross-docking data set but were subsequently defined by plotting the side chain χ-1 and χ-2 angle distributions for all binding site residues across a collection of more than 200 internal crystal structures.

**Fig. 2:**
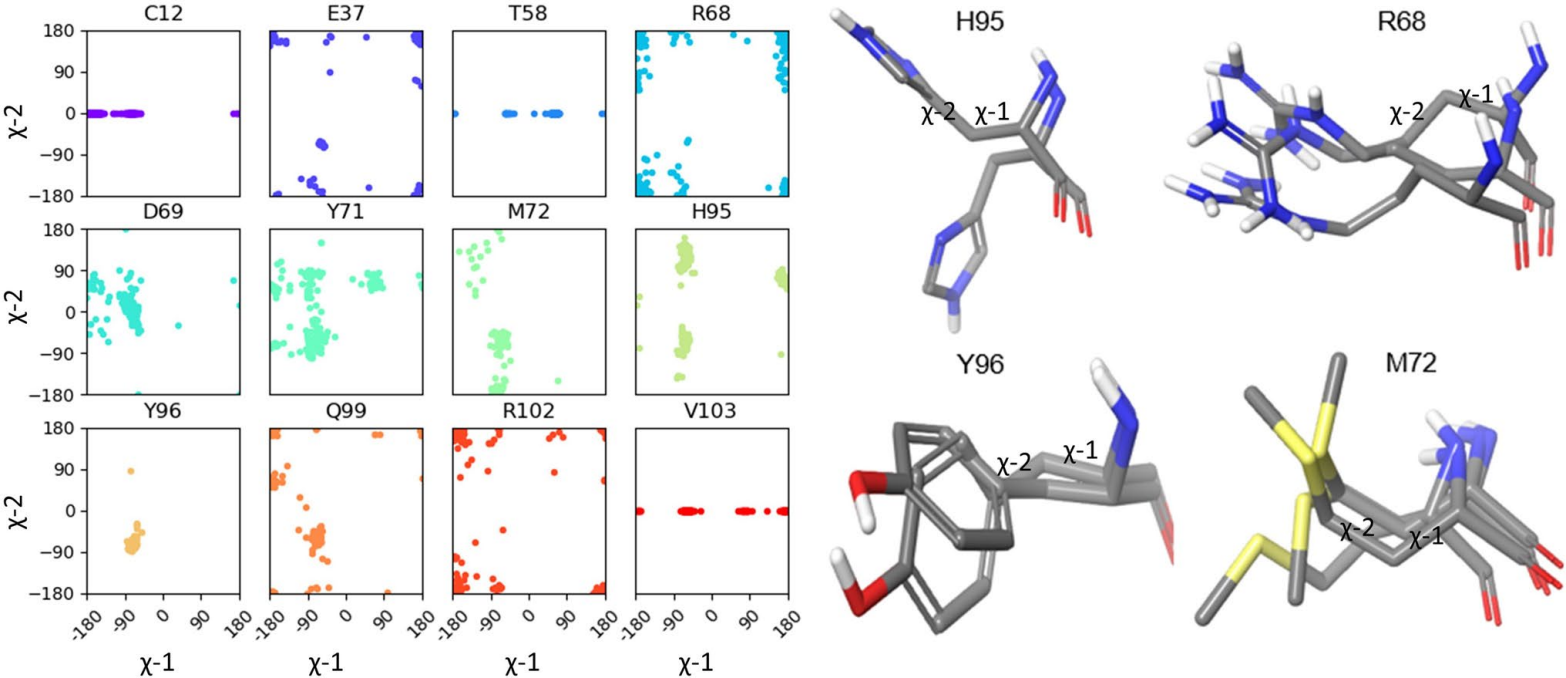
Side chain torsion angle distributions and representative structures for selected residues in the switch-II pocket. (Plots represent data from > 200 KRAS crystal structures.)

Figure 2 shows the torsional angle plots and representative structures for some of the selected residues. We determined the following 26 residues to have high degrees of conformational flexibility and thus to be key residues for conformational sampling and refinement in the FlexCovDock protocol (‘flexible residues’): Thr58–Thr74, Lys88, Asp92, His95, Tyr96, Gln99, Arg102, Val103, Cys12 and Glu37. Five of these residues (Glu62, Tyr64, Arg68, Met72 and His95) were determined to be capable of severely blocking the cross-docking of non-cognate ligands (‘blocking residues’) and were therefore mutated to alanine in the initial stage of FlexCovDock. The side chains of all flexible residues (including blocking residues) are repacked, and the backbone atoms are minimized in FlexCovDock workflow. This protocol limits the extent to which it is necessary to sample backbone conformational space. While incorporating loop prediction into the FlexCovDock protocol (e.g., using Prime loop prediction [26, 50]) could allow for larger switch-II loop movements, loop prediction is computationally expensive, and limited testing of such a workflow did not provide significantly improved docking accuracy.

### Binding pose prediction

**Fig. 3:**
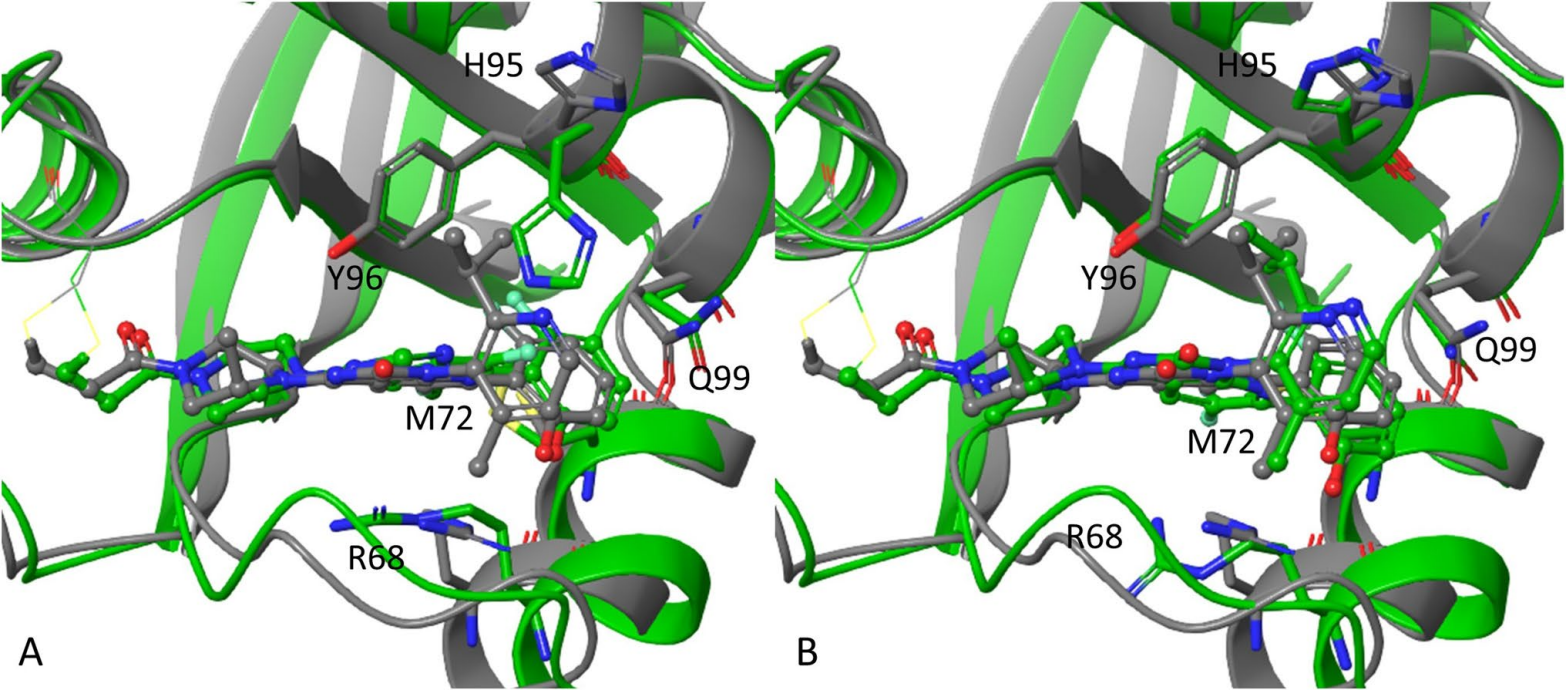
Comparison of the FlexCovDock-predicted binding modes of sotorasib (**3**) in the ARS-1620 (**2**) receptor and experimental structure. **A** superposition of the sotorasib (**3**; gray) and ARS-1620 (**2**; green) X-ray structures (PDB 6OIM & 5V9U, respectively). **B** predicted sotorasib pose using the ARS-1620 receptor (green, 0.8 Å RMSD) as compared with the crystallographically determined structure of sotorasib bound to KRAS^G12C^ (gray)

Figure 3 shows one example of the cross-docking of sotorasib (**3**) using the ARS-1620 (**2**) receptor structure (PDB 5V9U). In the ARS-1620 structure (Fig. 3A, green), the His95 side chain adopts a ‘closed’ conformation with the His95 side chain oriented toward the switch-II pocket, a conformation which would prevent the successful docking of sotorasib (**3**). In the FlexCovDock prediction (Fig. 3B, green), His95 was correctly moved to the ‘open’ conformation. As a result, the predicted top sotorasib (**3**) binding pose had only an 0.8 Å RMSD relative to the crystallographically observed binding pose. In contrast, the top binding pose identified using the conventional CovDock protocol demonstrated a 5.2 Å RMSD. Interestingly, except for His95, FlexCovDock did not predict all other side chains identically to their crystallographically determined positions (cf. Arg68, Fig. 3B), nor did it move the switch-II loop closer to the crystallographically determined position (cf. green and grey structures, Fig. 3B). It’s notable that relatively reasonable side chain conformational predictions alone were sufficient to enable correct ligand pose prediction—high precision side chain pose predictions were not required.

To further benchmark the performance of FlexCovDock, we collected ten co-crystal structures of structurally diverse ligands demonstrating conformationally diverse switch-II pocket poses. We then docked each ligand into every receptor structure, leading to a set of 100 docking simulations (10 self-docking and 90 cross-docking). (In the following paragraphs, we make no distinction between self- and cross-docking models, so ‘cross-docking’ should be understood to include these ten self-docking examples.) Comparing CovDock to the modified FlexCovDock protocol, in the 10 self-docking cases, CovDock correctly predicted the docked ligand pose to within a heavy atom RMSD of 2.0 Å in all cases. FlexCovDoc, by comparison, correctly predicted 9/10 ligand poses to within 2.0 Å RMSD and 10/10 poses to within 2.5 Å RMSD (see Supplementary Table S5 and S6).

**Fig. 4:**
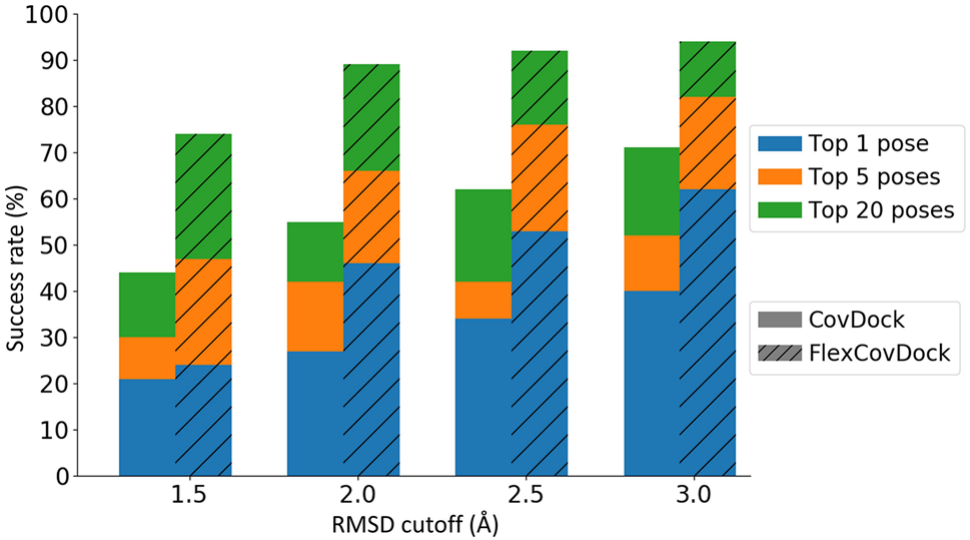
Comparison of the docking accuracy of CovDock and FlexCovDock using the cross-docking data set

Figure 4 compares the performance of CovDock and FlexCovDock across all 100 cross-docking jobs. As seen, FlexCovDock significantly improved the accuracy of binding pose prediction across a wide range of diffing RMSD cutoffs and ranking criteria. For example, using RMSD 2.0 Å as a cutoff and focusing on the top-ranked pose for each docking job, FlexCovDock correctly predicted the ligand pose in 46 cases, whereas CovDock was only successful in 27 cases. Focusing instead upon the best pose identified amongst the top 20 poses identify by each protocol, FlexCovDock correctly predicted the crystallographic binding pose 89% of the time, whereas CovDock was only successful 55% of the time.

### Prospective prediction in challenging cases

These 100 cross-docking cases were designed to model the very challenging case where a novel compound is identified which induces large structural change in a known receptor structure. Such cases can arise, for instance, when novel hit compounds are identified in a screening campaign or when new ligands emerging in the literature that are highly structurally distinct from historical ligands. As an illustration of the former situation, we have had the opportunity to test FlexCovDock prospectively on a set of four novel compounds whose X-ray crystal structures were only solved subsequently to modeling (internal data). In all four cases, the FlexCovDock-predicted ligand binding poses were predicted with less than 2.5 Å RMSD from the crystallographic pose and with average RMSD of 1.3 Å.

### Customization of flexible regions

FlexCovDock has also worked well in cases where only small changes were made to ligands with known co-crystal structure. One common issue with flexible receptor docking is that dramatic movements in receptor residues can be observed, leading to unexpected (and inaccurate) docking results. Despite allowing full receptor flexibility in the binding site, FlexCovDock has proven resistant to such challenges, working well across all ten of the aforementioned self-docking cases, thanks to the accurate side chain packing algorithm and energy function in Prime protein structure modeling. In practice, binding site flexibility can be customized for optimal performance in the FlexCovDock workflow by changing the list of ‘blocking residues’ and ‘flexible residues.’ For example, when focused changes are made to a specific region of a ligand, the flexible residue list can be tailored to include only those residues that interact with this region of the ligand. We have found that making such tailored changes can often lead to better pose prediction accuracy. The blocking residues can have substantial impact to the prediction accuracy. Supplementary Information Table S5 shows the results of using 4, 5, and 6 blocking residues in the cross-docking data set. We chose five blocking residues because it was the minimum set of residues that cover potential major side chain conformational changes across the docked ligands.

### Binding affinity prediction

**Fig. 5:**
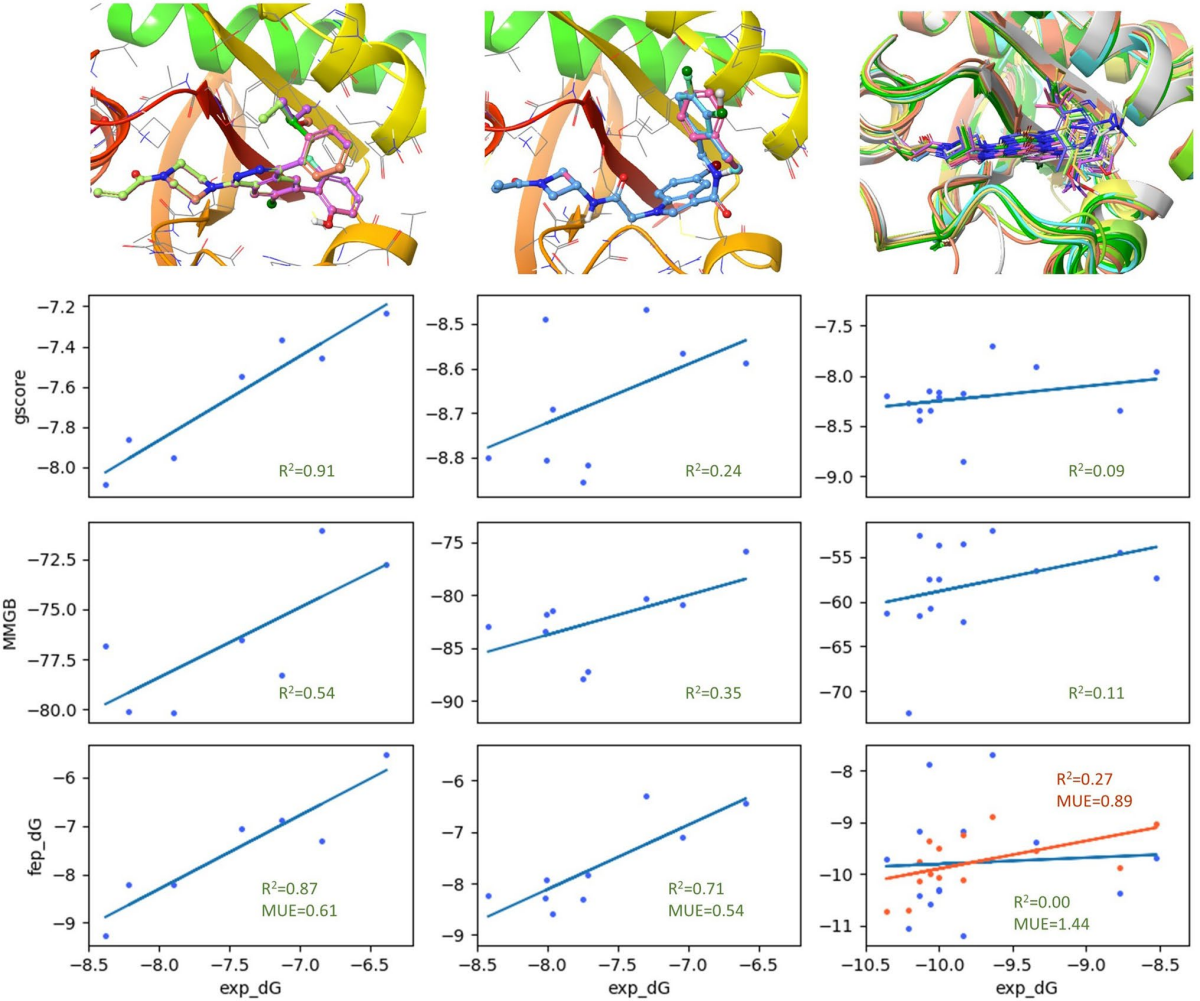
Overview of the binding modes of three group of compounds (*SAR1–3*) and their binding affinity predictions by docking score, MMGB, and FEP+. *SAR1* compounds (leftmost column) have the least ligand and receptor flexibility and are predicted well by all methods. (The top-left image shows the aligned compounds in the 6OIM crystal structure.) *SAR2* compounds (middle column) demonstrate significant ligand conformational flexibility but are bound to a common switch-II pocket pose. Binding affinities for these ligands are only predicted well by FEP+. (The top-middle image shows the aligned compounds in the 6P8X crystal structure.) *SAR3* compounds (rightmost column) induce conformational changes in the switch-II loop and are challenging to model by all methods. (The top-right image shows the superposition of all 14 cocrystal structures in the group.) Modification of FEP+ protocol (as described here) significantly improved the prediction accuracy for *SAR3* (orange correlation line)

As previously mentioned, the high conformational flexibility of the switch-II pocket also poses a great challenge to the accurate prediction of binding affinities. Here, we investigate the prediction of ligand binding affinities with three different methods, docking score, MMGB, and FEP+, using three group of compounds chosen to probe different portions of the switch-II pocket and to represent differing levels of ligand and receptor flexibility. Figure 5 shows these three groups of compounds (*SAR1*, *2*, & *3*) and their respective binding pockets.

*SAR1* and *SAR2* compounds make close receptor contacts within the His95 pocket [3]. *SAR1* comprises ligands that contact the β-carbon of the His95 side chain as well as the Tyr96 side chain. *SAR2*, in contrast, contains compounds that make broader contacts with the His95-pocket and engage in π-stacking interactions with the His95 side chain. *SAR3* compounds engage in direct interactions with residues in the switch-II loop. Based on the conformational flexibility of the switch-II pocket and the compounds within each *SAR* group, we can characterize these three *SAR* groups as ‘rigid receptor, rigid ligand’ (*SAR1*), ‘rigid receptor, flexible ligand’ (*SAR2*), and ‘flexible receptor, flexible ligand’ (*SAR3*), respectively.

Both docking score and MMGB calculations were performed using poses generated by the FlexCovDock protocol. *SAR1* and *SAR3* compounds were docked using the 6OIM crystal structure and *SAR2* compounds were docked using the 6P8X crystal structure, as these crystal structures contained the most similar ligands to these sets of compounds. For FEP+ calculations, simulations started from 6OIM (*SAR1* and *SAR3*) and 6P8X (*SAR2*); all ligands were structurally aligned to the crystallographic ligand.

As shown in Fig. 5, all binding affinity prediction methods perform well for *SAR1* (which represents a relatively easy case, given both rigid receptor and ligands). Although His95 can adopt both open- and closed-conformations, all compounds within this group bind to an ‘open’ His95 receptor pose. For the *SAR2* group, docking score and MMGB binding affinities have weak correlations with experimental binding affinities, however FEP+ still performs well, with excellent R^2^ and mean unsigned error (MUE). This, too, is understandable, given how the ligand flexibility of these *SAR2* compounds is handled by these methods. Scoring methods using a single, static structure such as docking and MMGB cannot capture conformational dynamics and entropic contributions to binding affinity, whereas FEP+ can encompass these factors by sampling the motion of these flexible ligands in their binding pocket.

For *SAR3*, all methods performed very poorly. This represents the most challenging case for binding energy prediction, as these ligands interact with a highly flexible receptor (switch-II loop), and induce multiple different conformations (as shown in crystal structures above). One interesting question is, can we obtain better results using the co-crystal structure of each individual ligand in the affinity scoring instead of the docked poses? FEP+ requires a single receptor structure in the calculation for the group of compounds, so it is unclear how to use multiple crystal structures. For docking score and MMGB calculations, scoring with each crystal structure did not show any improvement in the R^2^ values (data not shown).

### Slow convergence of FEP+ on switch-II loop conformational change

We suspected that the poor performance of FEP+ with *SAR3* compounds was due to difficulties in sampling switch-II loop conformations. One pair of compounds, compounds **11** and **12** from *SAR3* (compound 11 and 13 in SI Table S3, respectively), was chosen for a detailed study. The experimental relative binding affinity difference between these two compounds was 0.06 kcal/mol, however FEP+ predicted their relative difference in binding affinity (∆∆G) as − 2.52 kcal/mol using 6OIM receptor.

**Fig. 6:**
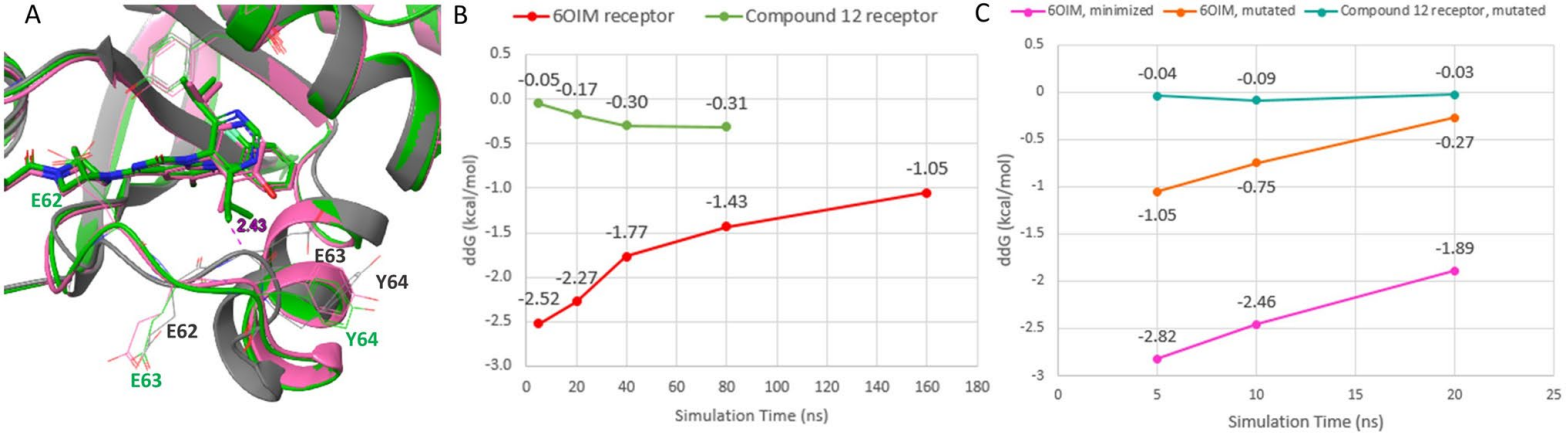
Switch-II loop conformational changes lead to slow convergence of FEP+; targeted protein mutations improve the accuracy. **A** The crystal structures of compound **11** (pink) and compound **12** (green). The 6OIM receptor structure is shown in gray and displays backbone atom clashes with both ligands. (Note: the positions of E63 and E62 in 6OIM are shifted relative to the corresponding residues in the compound **11** and **12** X-ray structures.) **B** and **C** FEP+ prediction of binding affinity differences (∆∆G) between compounds **11** and **12** using different receptor structures and simulation times. The experimental ∆∆G between compound **11** and **12** is 0.06 kcal/mol

As shown in Fig. 6A, in the 6OIM structure, Glu63 severely clashes with one isopropyl group in compound **12** in its binding pose extracted from its cognate crystal structure. Although compounds **11** and **12** have very similar switch-II loop conformations, these loop conformations differ significantly from that that observed in 6OIM. In the experimental X-ray structures of these two ligands, the backbone atom positions of residues Gln61, Glu62, and Glu63 are close to the positions of Glu62, Glu63, and Tyr64 of 6OIM, respectively. While this backbone conformational difference may appear small, it results in multiple backbone torsional angle changes that would take a very long time to access during an MD simulation.

Figure 6B illustrates FEP+ prediction of the binding affinity differences between compounds **11** and **12** using the 6OIM receptor (red line) and the crystallographically determined compound **12** receptor structure (green line). When simulation started using the compound **12** receptor structure (with correct switch-II loop conformation), FEP+ was able to accurately predict the experimental difference in binding affinity within 5 ns. (Longer simulation demonstrated that the MD trajectory had already converged.) Starting from the 6OIM receptor structure (where the switch-II loop initially clashes with compound **12**), the predicted binding affinity gradually improved with increasing simulation time, from − 2.52 kcal/mol at 5 ns to − 1.05 kcal/mol at 160 ns.

Although the study of this pair of compounds shows that FEP+ can achieve accurate binding affinity estimates and overcome incorrect starting conformations in the switch-II loop, doing so requires very long simulation times, making this brute-force approach impractical both in terms of computing cost and turnaround times.

### Loop mutation strategy

To overcome these issues, we developed a new strategy that both significantly accelerated the convergence of FEP+ calculations while also achieving enhanced accuracy. In this strategy, we mutated residues Glu62, Glu63, Tyr64 and Glu76 to glycine and used the mutated receptor structure in the FEP+ calculation. The rationale was as follows: (1) with these mutations, the backbone conformational transition in MD would be much faster, and (2) these residues do not have side chain contact with the ligands; thus, these mutations would not have large impacts on the relative binding affinity of these ligands. Note that Glu76 is at the end of α2-helix and does not belong to switch-II pocket. The mutation of glutamate 76 to glycine (E76G) created a segment of 3 consecutive glycines (with Gly75 and Gly77), which increased the mobility of the α2-helix. This mutation was not necessary for this specific pair of compounds **11** and **12** (see Supplementary Information Table S7), but we included it in the mutated structure because it was beneficial when the ligands caused significant movement of α2-helix as we encountered in our internal project.

Figure 6C shows the improved FEP+ accuracy with faster convergence using this loop mutation strategy. Using the mutated 6OIM receptor, 5 ns FEP+ simulation already showed a greatly improved predicted ∆∆G (− 1.05 vs. − 2.82 kcal/mol), and 20 ns of FEP+ further improved the ∆∆G to − 0.27 kcal/mol (orange line). Comparing the resulting ∆∆G to that obtained using the native compound **12** receptor structure (− 0.03 kcal/mol, green line), it is apparent that this mutation strategy did not significantly alter the calculated binding affinity, as expected.

To investigate the necessity of proper conformational sampling of the switch-II loop in generating an accurate binding affinity calculation, we also examined minimization of the 6OIM structure while holding the aligned compound **12** fixed in the pocket prior to FEP+ calculation. The results obtained using the minimized 6OIM receptor were not much better than those obtained using the original (unminimized) 6OIM structure (purple line, Fig. 6C). This showed that a simple minimization to relieve the steric clash between ligand and the receptor was insufficient to improve binding energy predictions, and that the proper backbone conformational transition was required for accurate FEP+ binding energy prediction. Finally, we used the mutated 6OIM structure for FEP+ calculations for all 14 compounds in *SAR3*. Using this approach, the MUE was improved from 1.44 to 0.89 kcal/mol, as shown in Fig. 5.

## Conclusions

The high receptor flexibility of the KRAS switch-II pocket presents a challenge to current state-of-the-art methods for binding pose and binding affinity prediction. In this work, we report a modification of the CovDock workflow to incorporate receptor flexibility, enabling the accurate binding pose prediction of ligands which induce large conformational changes in the ligand binding site. We have validated this protocol using a large cross-docking data set and have used this methodology to successfully optimize multiple switch-II pocket-targeted inhibitors of KRAS^G12C^. Although we illustrate this protocol in the refinement of covalent inhibitors for KRAS^G12C^, it should be noted that this methodology should also prove extensible to the modeling of covalent inhibitors of other flexible proteins.

We additionally report refinements to FEP+ based binding affinity prediction methods that facilitate binding energy predictions with conformationally flexible receptors. In the case of KRAS^G12C^ inhibitor binding affinity prediction, we show that compounds that directly engage the conformationally dynamic switch-II loop pose the greatest challenge. Structurally diverse inhibitors can induce differing switch-II loop conformations that FEP+ can take a very long time to sample. We have shown that mutating a small number of residues within and near the switch-II loop can greatly improve the accuracy of FEP+ without requiring significantly lengthened simulation time. This strategy has its limitations, however, and relies on the assumption that the side chains of the mutated residues do not make strong contacts with the ligands. Caution should be exercised when this assumption may be in doubt.

Receptor flexibility presents a major challenge for the accurate forecasting of protein–ligand binding for many pharmaceutically relevant targets. The efficient sampling of protein backbone conformational changes and accurate calculation of protein reorganization energies remain an important unsolved problem for the future research. The strategy presented in this work to address the conformational flexibility of switch-II loop, although not a universal solution, should prove useful in other similar situations.

## Supplementary Information

Below is the link to the electronic supplementary material.

Supplementary file 1 (DOCX 331 KB)—Schematic workflow of FlexCovDock and CovDock; Structures and experimental binding affinities for SAR1–3 compounds; crystallographic data collection and refinement statistics; CovDock and FlexCov-Dock cross-docking results. FEP+  prediction results using various loop mutations.

## Data Availability

All the data generated in this work is freely available in the supplementary information or by contacting the corresponding author. Three new crystal structures, 8DNI, 8DNJ, and 8DNK, have been deposited to PDB.

## Abbreviations

KRAS: Kirsten rat sarcoma virus oncogene
GDP: Guanosine diphosphate
GPU: Graphics processing unit
GTP: Guanosine triphosphate
API: Application programming interface
FEP: Free energy perturbation
MD: Molecular dynamics
MMGB: Molecular mechanics generalized Born approximation
MMGBSA: Molecular mechanics generalized Born surface area
ns: Nanosecond
RMSD: Root mean squared deviation
MUE: Mean unsigned error
SAR: Structure–activity relationship
TI: Thermodynamic integration
