# Supplementary material for: Modeling receptor flexibility in the structure-based design of KRASG12C inhibitors
Source: J Comput Aided Mol Des. 2022 Aug 5;36(8):591–604. doi: 10.1007/s10822-022-00467-0 (PMC9512760; doi:10.1007/s10822-022-00467-0)
Supplement: Supplementary file 1 — (DOCX 331 KB)—Schematic workflow of FlexCovDock and CovDock; Structures and experimental binding affinities for SAR1–3 compounds; crystallographic data collection and refinement statistics; CovDock and FlexCov-Dock cross-docking results. FEP+ prediction results using various loop mutations. [file 10822_2022_467_MOESM1_ESM.docx]

SUPPLEMENTARY INFORMATION

Modeling Receptor Flexibility in the Structure-Based Design of KRAS^G12C^ Inhibitors

Kai Zhu,^†,*^ Cui Li,^‡^ Kingsley Y. Wu,^§^ Christopher Mohr,^†^ Xun Li,^‡^ Brian Lanman^‖^

^†^ Department of Molecular Engineering, ^‖^ Department of Medicinal Chemistry, Amgen Inc., One Amgen Center Drive, Thousand Oaks, California 91320, USA
^‡^ Amgen Asia R&D Center, 13th Floor, Building No. 2, 4560 Jinke Road, Zhangjiang, Shanghai, 201210, China
^§^ Department of Chemistry, University of California, Riverside, 501 Big Springs Road, CA 92521, USA


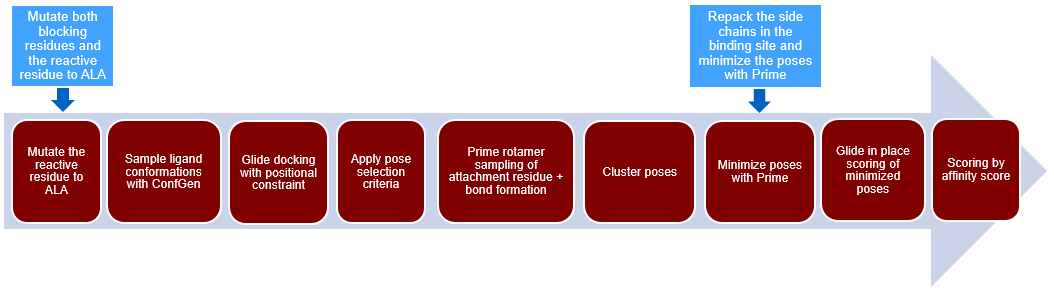


Fig. S1. Schematic comparison of FlexCovDock and CovDock workflow. Maroon squares are the steps in CovDock. Blue squares are modifications in FlexCovDock to incorporate receptor flexibility.

Table S1. *SAR1* compounds: structures and experimental binding affinities. Data from Lanman, et. al., Table 1.^1^ (Compound IDs taken from the original table.)

| Compound |  |  |  |
| --- | --- | --- | --- |
|  | **2** | **3** | **4** |
| ΔG (kcal/mol) | -6.38 | -7.13 | -7.41 |
| Compound |  |  |  |
|  | **5** | **6** | **7** |
| ΔG (kcal/mol) | -8.21 | -6.85 | -7.90 |
| Compound |  |  |  |
|  | **8** |  |  |
| ΔG (kcal/mol) | -8.38 |  |  |

**Table S2.** *SAR2* compounds: structures and experimental binding affinities. Data from Cee, e.t al., Table 1.^2^ (Compound IDs taken from the original table.)

| Compound |  |  |  |
| --- | --- | --- | --- |
|  | **6** | **7** | **8** |
| ΔG (kcal/mol) | -8.15 | -6.59 | -7.30 |
| Compound |  |  |  |
|  | **9** | **10** | **11** |
| ΔG (kcal/mol) | -7.04 | -8.01 | -7.97 |
| Compound |  |  |  |
|  | **12** | **13** | **14** |
| ΔG (kcal/mol) | -7.71 | -7.74 | -8.00 |

Table S3. *SAR3* compounds: structures and experimental binding affinities.

| Compound |  |  |  |  |
| --- | --- | --- | --- | --- |
|  | **1** | **2** | **3** | **4** |
| ΔG (kcal/mol) | -10.00 | -10.21 | -9.34 | -8.52 |
| Compound |  |  |  |  |
|  | **5** | **6** | **7** | **8** |
| ΔG (kcal/mol) | -8.77 | -10.06 | -10.13 | -10.36 |
| Compound |  |  |  |  |
|  | **9** | **10** | **11** | **12** |
| ΔG (kcal/mol) | -9.64 | -9.84 | -10.13 | -9.84 |
| Compound |  |  |  |  |
|  | **13** | **14** |  |  |
| ΔG (kcal/mol) | -10.07 | -10.00 |  |  |

**Table S4.** Crystallographic data collection and refinement statistics.

|  | **8DNI** (Compound **8**) | **8DNJ** (Compound **9**) | **8DNK** (Compound **10**) |
| --- | --- | --- | --- |
| **Data collection^*^**  Wavelength (Å) | 1.00000 | 0.99999 | 0.99999 |
| Space group | P 65 2 2 | P 1 21 1 | P 2 3 |
| Cell dimensions |  |  |  |
| *a*, *b*, *c* (Å) | 41.20, 41.20, 348.50 | 37.59, 112.16, 57.82 | 91.18, 91.18, 91.18 |
| α, β, γ (°) | 90.00, 90.00, 120.00 | 90.00, 96.80, 90.00 | 90.00, 90.00, 90.00 |
| Resolution (Å) | 30.0–1.50 (1.55–1.50) | 112.11–1.81 (1.84–1.81) | 64.48–2.23 (2.27–2.23) |
| Unique reflections | 30081 | 43236 | 12603 |
| R_merge_ | 0.118 (0.885) | 0.215 (1.557) | 0.337 (2.801) |
| *I* / σ | 8.1 (5.0) | 4.8 (1.1) | 11.8 (2.4) |
| *CC half* | 1.016 (0.936) | 0.948 (0.564) | 0.998 (0.757) |
| Completeness (%) | 100.0 (100.0) | 99.8 (95.2) | 100.0 (100.0) |
| Redundancy | 31.0 (30.7) | 6.3 (5.3) | 38.4 (40.1) |
|  |  |  |  |
| **Refinement** |  |  |  |
| Resolution (Å) | 30.00 – 1.50 | 30.00 – 1.81 | 30.00 – 2.23 |
| Completeness (%) | 97.67 | 99.46 | 99.88 |
| No. reflections | 27784 | 40836 | 11916 |
| *R*_work_ / *R*_free_ | 0.2411 / 0.2539 | 0.2392 / 0.2889 | 0.1809 / 0.2354 |
| No. atoms | 1585 | 4418 | 1469 |
| Protein | 1370 | 4042 | 1359 |
| Ligand/ion | 64 | 177 | 68 |
| Water | 151 | 199 | 42 |
| Average B overall (Å^2^) | 13.74 | 25.834 | 39.26 |
| R.m.s. deviations |  |  |  |
| Bond lengths (Å) | 0.005 | 0.003 | 0.013 |
| Bond angles (°) | 1.192 | 1.323 | 1.851 |
| Ramachandran ^ |  |  |  |
| Favored (%) | 98.8 | 98.2 | 98.2 |
| Allowed (%) | 1.2 | 1.8 | 1.8 |
| Outliers (%) | 0 | 0 | 0 |
|  |  |  |  |

One crystal data set was collected for each of these structures

^*^ Values in parentheses are for highest-resolution shell.

^ MolProbity Ramachandran *Lovell, Davis, et al. Proteins 50:437 (2003)*

Table S5. FlexCovDock cross-docking results using different set of blocking residues. The 5 blocking residues are chosen as the default protocol.

| Blocking residues |  | #<1.5 Å | #<2.0 Å | #<2.5 Å | #<3.0 Å |
| --- | --- | --- | --- | --- | --- |
| H95, M72, R68,  E64 | Top1 | 16 | 34 | 44 | 54 |
|  | Top5 | 38 | 56 | 71 | 77 |
|  | Top20 | 67 | 77 | 83 | 89 |
| H95, M72, R68,  E64, E62 | Top1 | 24 | 46 | 53 | 62 |
|  | Top5 | 47 | 66 | 76 | 82 |
|  | Top20 | 74 | 89 | 92 | 94 |
| H95, M72, R68,  E64, E62, Q99 | Top1 | 26 | 43 | 51 | 57 |
|  | Top5 | 48 | 71 | 79 | 88 |
|  | Top20 | 75 | 86 | 92 | 94 |

Table S6. CovDock and FlexCovDock cross-docking results: rmsd (Å) and accuracy count.

| **CovDock** |  |  |  |  |  |  |  |  |  |  |  |  |  |  |
| --- | --- | --- | --- | --- | --- | --- | --- | --- | --- | --- | --- | --- | --- | --- |
| **Top 1** | **5F2E** | **5V9U** | **6OIM** | **6P8X** | **6UT0** | **6T5B** | **6TAN** | **8DNI** | **8DNJ** | **8DNK** | **#<1.5,** | **#<2.0,** | **#<2.5,** | **#<3.0** |
| **5F2E** | 0.3 | 3.4 | 9.3 | 10.7 | 6.5 | 14.8 | 2.5 | 8.8 | 10.3 | 6.4 | 1 | 1 | 2 | 2 |
| **5V9U** | 5.4 | 0.4 | 5.2 | 3.8 | 3.4 | 5.2 | 1.8 | 3.1 | 1.0 | 6.8 | 2 | 3 | 3 | 3 |
| **6OIM** | 3.4 | 1.0 | 0.4 | 2.8 | 1.7 | 1.6 | 5.5 | 5.4 | 7.0 | 7.4 | 2 | 4 | 4 | 5 |
| **6P8X** | 4.8 | 4.2 | 1.5 | 0.4 | 1.3 | 5.5 | 2.4 | 5.3 | 3.5 | 7.3 | 3 | 3 | 4 | 4 |
| **6UT0** | 2.1 | 1.2 | 8.9 | 7.5 | 1.0 | 1.8 | 2.3 | 8.0 | 10.3 | 2.0 | 2 | 3 | 6 | 6 |
| **6T5B** | 10.4 | 0.6 | 7.1 | 5.7 | 8.8 | 0.3 | 1.8 | 6.2 | 7.4 | 7.9 | 2 | 3 | 3 | 3 |
| **6TAN** | 4.5 | 4.1 | 13.1 | 4.7 | 9.7 | 13.6 | 0.4 | 9.0 | 4.0 | 5.1 | 1 | 1 | 1 | 1 |
| **8DNI** | 4.9 | 1.1 | 4.8 | 2.1 | 2.7 | 3.0 | 5.1 | 0.5 | 3.7 | 5.3 | 2 | 2 | 3 | 5 |
| **8DNJ** | 4.1 | 0.8 | 7.7 | 3.5 | 2.6 | 1.3 | 1.3 | 12.5 | 0.3 | 9.8 | 4 | 4 | 4 | 5 |
| **8DNK** | 1.4 | 2.9 | 7.4 | 3.7 | 1.9 | 6.7 | 2.5 | 2.8 | 3.5 | 0.4 | 2 | 3 | 4 | 6 |
|  |  |  |  |  |  |  |  |  |  |  | 21 | 27 | 34 | 40 |
| **Top 5** | **5F2E** | **5V9U** | **6OIM** | **6P8X** | **6UT0** | **6T5B** | **6TAN** | **8DNI** | **8DNJ** | **8DNK** | **#<1.5,** | **#<2.0,** | **#<2.5,** | **#<3.0** |
| **5F2E** | 0.3 | 2.9 | 7.1 | 8.0 | 6.5 | 14.8 | 1.2 | 8.8 | 2.7 | 1.3 | 3 | 3 | 3 | 5 |
| **5V9U** | 3.2 | 0.3 | 4.8 | 3.8 | 1.3 | 4.5 | 1.7 | 2.0 | 0.8 | 6.6 | 3 | 4 | 5 | 5 |
| **6OIM** | 3.4 | 0.8 | 0.3 | 2.8 | 1.7 | 1.5 | 2.1 | 5.4 | 4.9 | 6.4 | 2 | 4 | 5 | 6 |
| **6P8X** | 4.4 | 0.5 | 1.0 | 0.4 | 1.3 | 5.2 | 1.8 | 1.2 | 3.3 | 4.5 | 5 | 6 | 6 | 6 |
| **6UT0** | 1.2 | 1.1 | 8.7 | 3.4 | 1.0 | 1.8 | 1.7 | 8.0 | 7.6 | 2.0 | 3 | 5 | 6 | 6 |
| **6T5B** | 8.2 | 0.6 | 7.1 | 3.4 | 1.7 | 0.2 | 1.8 | 6.2 | 7.2 | 7.9 | 2 | 4 | 4 | 4 |
| **6TAN** | 4.3 | 4.0 | 12.3 | 3.7 | 9.3 | 7.9 | 0.2 | 9.0 | 1.4 | 5.1 | 2 | 2 | 2 | 2 |
| **8DNI** | 4.0 | 1.1 | 4.8 | 2.1 | 1.3 | 2.0 | 5.0 | 0.3 | 0.8 | 5.3 | 4 | 5 | 6 | 6 |
| **8DNJ** | 3.8 | 0.7 | 6.8 | 3.1 | 2.6 | 1.3 | 1.3 | 6.5 | 0.3 | 6.6 | 4 | 4 | 4 | 5 |
| **8DNK** | 0.7 | 2.6 | 7.2 | 3.4 | 1.8 | 6.2 | 1.8 | 1.8 | 2.7 | 0.4 | 2 | 5 | 5 | 7 |
|  |  |  |  |  |  |  |  |  |  |  | 30 | 42 | 46 | 52 |
| **Top 20** | **5F2E** | **5V9U** | **6OIM** | **6P8X** | **6UT0** | **6T5B** | **6TAN** | **8DNI** | **8DNJ** | **8DNK** | **#<1.5,** | **#<2.0,** | **#<2.5,** | **#<3.0** |
| **5F2E** | 0.3 | 2.7 | 7.0 | 6.1 | 6.5 | 9.2 | 0.9 | 4.9 | 2.4 | 1.1 | 3 | 3 | 4 | 5 |
| **5V9U** | 2.2 | 0.3 | 4.8 | 2.0 | 1.3 | 4.5 | 1.1 | 2.0 | 0.8 | 1.9 | 4 | 6 | 8 | 8 |
| **6OIM** | 2.5 | 0.8 | 0.3 | 0.8 | 1.7 | 1.5 | 1.6 | 2.1 | 4.5 | 4.1 | 3 | 6 | 7 | 8 |
| **6P8X** | 2.1 | 0.5 | 1.0 | 0.4 | 1.0 | 5.2 | 0.5 | 1.2 | 2.7 | 4.1 | 6 | 6 | 7 | 8 |
| **6UT0** | 1.2 | 0.9 | 6.7 | 3.2 | 0.7 | 1.8 | 0.5 | 6.8 | 1.3 | 1.4 | 6 | 7 | 7 | 7 |
| **6T5B** | 2.0 | 0.6 | 7.1 | 2.6 | 1.7 | 0.2 | 0.7 | 6.2 | 1.4 | 7.9 | 4 | 5 | 6 | 7 |
| **6TAN** | 3.2 | 1.8 | 9.0 | 3.2 | 8.9 | 7.9 | 0.2 | 9.0 | 1.4 | 4.5 | 2 | 3 | 3 | 3 |
| **8DNI** | 2.6 | 0.7 | 1.4 | 0.7 | 1.2 | 1.3 | 1.4 | 0.3 | 0.8 | 5.3 | 8 | 8 | 8 | 9 |
| **8DNJ** | 2.3 | 0.7 | 3.8 | 2.2 | 2.2 | 1.3 | 1.3 | 2.1 | 0.3 | 4.5 | 4 | 4 | 8 | 8 |
| **8DNK** | 0.7 | 1.8 | 7.2 | 2.4 | 1.4 | 4.2 | 0.9 | 1.8 | 1.8 | 0.4 | 4 | 7 | 8 | 8 |
|  |  |  |  |  |  |  |  |  |  |  | 44 | 55 | 66 | 71 |
| **FlexCovDock** |  |  |  |  |  |  |  |  |  |  |  |  |  |  |
| **Top 1** | **5F2E** | **5V9U** | **6OIM** | **6P8X** | **6UT0** | **6T5B** | **6TAN** | **8DNI** | **8DNJ** | **8DNK** | **#<1.5,** | **#<2.0,** | **#<2.5,** | **#<3.0** |
| **5F2E** | 0.9 | 3.2 | 5.0 | 6.0 | 1.0 | 1.8 | 2.1 | 0.7 | 3.1 | 1.7 | 3 | 5 | 6 | 6 |
| **5V9U** | 2.6 | 1.6 | 2.4 | 3.5 | 1.6 | 2.4 | 4.3 | 2.1 | 3.8 | 6.6 | 0 | 2 | 5 | 6 |
| **6OIM** | 3.2 | 0.7 | 1.1 | 2.5 | 1.4 | 5.3 | 1.5 | 5.1 | 3.1 | 7.4 | 3 | 4 | 4 | 5 |
| **6P8X** | 1.3 | 0.5 | 2.8 | 2.1 | 1.1 | 1.6 | 1.8 | 1.3 | 3.7 | 2.0 | 4 | 7 | 8 | 9 |
| **6UT0** | 0.5 | 1.2 | 2.9 | 7.1 | 1.1 | 2.7 | 1.5 | 1.0 | 2.9 | 8.1 | 5 | 5 | 5 | 8 |
| **6T5B** | 4.3 | 0.9 | 2.3 | 2.0 | 7.5 | 0.8 | 1.9 | 4.3 | 0.8 | 4.9 | 3 | 5 | 6 | 6 |
| **6TAN** | 2.0 | 3.4 | 5.1 | 5.9 | 0.6 | 4.9 | 0.5 | 2.9 | 4.2 | 4.8 | 2 | 3 | 3 | 4 |
| **8DNI** | 2.5 | 1.8 | 1.4 | 2.6 | 7.3 | 1.9 | 4.7 | 0.6 | 3.8 | 8.0 | 2 | 4 | 4 | 6 |
| **8DNJ** | 3.3 | 1.5 | 5.0 | 6.7 | 1.9 | 1.9 | 1.8 | 2.1 | 0.6 | 3.6 | 1 | 5 | 6 | 6 |
| **8DNK** | 1.6 | 3.3 | 5.0 | 5.4 | 1.6 | 1.7 | 1.9 | 1.7 | 3.5 | 0.6 | 1 | 6 | 6 | 6 |
|  |  |  |  |  |  |  |  |  |  |  | 24 | 46 | 53 | 62 |
| **Top 5** | **5F2E** | **5V9U** | **6OIM** | **6P8X** | **6UT0** | **6T5B** | **6TAN** | **8DNI** | **8DNJ** | **8DNK** | **#<1.5,** | **#<2.0,** | **#<2.5,** | **#<3.0** |
| **5F2E** | 0.4 | 0.8 | 4.8 | 6.0 | 0.7 | 0.8 | 1.9 | 0.7 | 1.7 | 1.6 | 5 | 8 | 8 | 8 |
| **5V9U** | 2.2 | 0.5 | 0.8 | 1.8 | 0.8 | 0.6 | 4.3 | 1.6 | 1.6 | 1.8 | 4 | 8 | 9 | 9 |
| **6OIM** | 2.7 | 0.6 | 1.1 | 1.9 | 1.4 | 1.9 | 1.5 | 2.9 | 2.2 | 4.1 | 3 | 6 | 7 | 9 |
| **6P8X** | 1.3 | 0.5 | 1.6 | 1.1 | 1.1 | 0.8 | 1.7 | 1.3 | 3.4 | 1.4 | 7 | 9 | 9 | 9 |
| **6UT0** | 0.5 | 0.8 | 2.3 | 2.2 | 1.1 | 1.4 | 0.8 | 1.0 | 2.9 | 1.7 | 6 | 7 | 9 | 10 |
| **6T5B** | 3.6 | 0.8 | 1.8 | 1.5 | 2.3 | 0.3 | 1.7 | 2.5 | 0.8 | 4.9 | 3 | 6 | 8 | 8 |
| **6TAN** | 1.8 | 2.2 | 4.9 | 4.7 | 0.6 | 4.7 | 0.3 | 1.6 | 3.8 | 2.0 | 2 | 4 | 6 | 6 |
| **8DNI** | 2.5 | 0.7 | 1.4 | 2.6 | 6.4 | 1.3 | 1.3 | 0.5 | 0.8 | 4.6 | 6 | 6 | 6 | 8 |
| **8DNJ** | 3.0 | 0.7 | 4.8 | 1.4 | 1.5 | 1.3 | 1.4 | 2.1 | 0.5 | 2.7 | 6 | 6 | 7 | 8 |
| **8DNK** | 1.0 | 2.2 | 5.0 | 3.0 | 1.6 | 1.0 | 1.0 | 1.0 | 3.2 | 0.6 | 5 | 6 | 7 | 7 |
|  |  |  |  |  |  |  |  |  |  |  | 47 | 66 | 76 | 82 |
| **Top 20** | **5F2E** | **5V9U** | **6OIM** | **6P8X** | **6UT0** | **6T5B** | **6TAN** | **8DNI** | **8DNJ** | **8DNK** | **#<1.5,** | **#<2.0,** | **#<2.5,** | **#<3.0** |
| **5F2E** | 0.4 | 0.8 | 4.7 | 1.4 | 0.7 | 0.8 | 1.5 | 0.7 | 0.7 | 1.6 | 7 | 9 | 9 | 9 |
| **5V9U** | 0.9 | 0.4 | 0.8 | 1.2 | 0.8 | 0.6 | 1.2 | 1.5 | 0.7 | 1.6 | 9 | 10 | 10 | 10 |
| **6OIM** | 1.7 | 0.6 | 0.5 | 0.8 | 1.4 | 1.2 | 1.5 | 1.8 | 0.5 | 2.7 | 6 | 9 | 9 | 10 |
| **6P8X** | 0.9 | 0.5 | 1.0 | 1.0 | 1.1 | 0.8 | 0.7 | 1.3 | 1.0 | 1.4 | 10 | 10 | 10 | 10 |
| **6UT0** | 0.5 | 0.6 | 1.3 | 0.9 | 0.7 | 0.7 | 0.7 | 1.0 | 0.6 | 1.7 | 9 | 10 | 10 | 10 |
| **6T5B** | 3.2 | 0.8 | 1.8 | 0.8 | 1.8 | 0.3 | 1.1 | 1.4 | 0.8 | 4.7 | 6 | 8 | 8 | 8 |
| **6TAN** | 1.0 | 1.6 | 4.7 | 3.6 | 0.6 | 4.7 | 0.3 | 1.6 | 0.6 | 1.5 | 4 | 7 | 7 | 7 |
| **8DNI** | 0.9 | 0.6 | 1.4 | 1.4 | 1.2 | 1.3 | 0.9 | 0.5 | 0.5 | 2.1 | 9 | 9 | 10 | 10 |
| **8DNJ** | 2.0 | 0.5 | 2.5 | 1.4 | 1.0 | 1.2 | 1.2 | 1.3 | 0.5 | 2.6 | 7 | 7 | 9 | 10 |
| **8DNK** | 1.0 | 0.9 | 1.7 | 1.5 | 1.6 | 0.8 | 0.6 | 0.7 | 1.0 | 0.6 | 7 | 10 | 10 | 10 |
|  |  |  |  |  |  |  |  |  |  |  | 74 | 89 | 92 | 94 |

Table S7. FEP+ prediction of relative binding affinity ∆∆G between compound 11 and 12 using various mutations on 6OIM. The experimental ∆∆G (11->12) is 0.06 kcal/mol.

| Loop mutations | Simulation time (nanosecond) | ∆∆G (kcal/mol) |
| --- | --- | --- |
| No mutation | 10 | -2.24 |
|  | 20 | -2.27 |
| E62G, E63G, Y64G | 10 | -0.76 |
|  | 20 | -0.28 |
| E62G, E63G, Y64G, E76G | 10 | -0.75 |
|  | 20 | -0.27 |
| E62G, E63G, E76G | 10 | -1.72 |
|  | 20 | -1.05 |

References

1. Lanman BA, Allen JR, Allen JG, Amegadzie AK, Ashton KS, Booker SK, Chen JJ, Chen N, Frohn MJ, Goodman G, Kopecky DJ, Liu L, Lopez P, Low JD, Ma V, Minatti AE, Nguyen TT, Nishimura N, Pickrell AJ, Reed AB, Shin Y, Siegmund AC, Tamayo NA, Tegley CM, Walton MC, Wang HL, Wurz RP, Xue M, Yang KC, Achanta P, Bartberger MD, Canon J, Hollis LS, McCarter JD, Mohr C, Rex K, Saiki AY, San Miguel T, Volak LP, Wang KH, Whittington DA, Zech SG, Lipford JR, Cee VJ (2020) Discovery of a Covalent Inhibitor of KRAS(G12C) (AMG 510) for the Treatment of Solid Tumors. J Med Chem 63: 52–65.

2. Shin Y, Jeong JW, Wurz RP, Achanta P, Arvedson T, Bartberger MD, Campuzano IDG, Fucini R, Hansen SK, Ingersoll J, Iwig JS, Lipford JR, Ma V, Kopecky DJ, McCarter J, San Miguel T, Mohr C, Sabet S, Saiki AY, Sawayama A, Sethofer S, Tegley CM, Volak LP, Yang K, Lanman BA, Erlanson DA, Cee VJ (2019) Discovery of N-(1-Acryloylazetidin-3-yl)-2-(1H-indol-1-yl)acetamides as Covalent Inhibitors of KRAS(G12C). ACS Med Chem Lett 10: 1302–1308.

3. Lovell SC, Davis IW, Arendall WB, de Bakker PI, Word JM, Prisant MG, Richardson JS, Richardson DC (2003) Structure validation

by Calpha geometry: phi,psi and Cbeta deviation. Proteins 50: 437–450.
